# Minimally Invasive Video-Assisted Surgical Management for Parapharyngeal Metastases From Papillary Thyroid Carcinoma: A Case Series Report

**DOI:** 10.3389/fonc.2019.01226

**Published:** 2019-11-22

**Authors:** Shi-Tong Yu, Wan-Zhi Chen, De-Bin Xu, Rong Xie, Tao Zhou, Ji-Chun Yu

**Affiliations:** ^1^Department of Thyroid Surgery, The Second Affiliated Hospital of Nanchang University, Nanchang, China; ^2^Department of General Surgery, Nanfang Hospital, Southern Medical University, Guangzhou, China

**Keywords:** minimally invasive video-assisted technique, parapharyngeal lymph node metastases, thyroid cancer, parapharyngeal space, endoscopic surgery

## Abstract

**Background:** Papillary thyroid carcinoma (PTC) is the most prevalent cancer type in the endocrine system. Metastases to parapharyngeal lymph nodes (PPLNs) are rare. Herein, we reported a case series of PTC patients with PPLN metastases operated on by using the minimally invasive video-assisted (MIVA) technique to evaluate the safety and effectiveness of this technique.

**Method:** In this single-institutional study, six consecutive PTC patients with PPLN metastases between January 2012 and July 2018 were enrolled. All PPLNs were managed by the MIVA technique.

**Result:** Six patients (three women and three men) who underwent surgery were enrolled in the current study. The median age of patients was 40.5 years (39–66). Five patients (83.3%) were diagnosed with primary PTC with PPLN metastases, and one patient had PTC recurrence in the PPLNs 17 years after her first PTC surgery. Surgical treatment was successful in all patients, and the median operative time and bleeding volume were 185 (100–280) min and 85 (30–120) ml, respectively. None of the patients experienced post-operative complications except for one patient who experienced dysphagia, which resolved within 3 months. During a median follow-up of 15 months (10–31), none of the patients exhibited recurrence or persistent disease.

**Conclusion:** The MIVA transcervical approach was technically feasible and reliable, with less invasiveness for PTC patients with PPLN metastases. Future studies are needed to accumulate more experience, investigate the indications of the technique, and determine the long-term oncological safety.

## Introduction

Papillary thyroid carcinoma (PTC) is the most prevalent cancer type in the endocrine system, with an increasing incidence worldwide over the past decade. The cervical lymph node metastasis rate ranges from 30 to 80% from the first clinic visit (1). Metastases to parapharyngeal lymph nodes (PPLNs) are rare. To the best of our knowledge, <100 cases of PPLN metastases were reported in the literature (2–6), with metastasis rates ranging from 0.43 to 2.5%.

The parapharynegal space (PPS) is an inverted pyramid anatomical region that comprises the skull base, with its apex reaching the greater cornu of the hyoid bone. The PPS is divided into pre-styloid and post-styloid compartments by thick layers of fascia extending from the styloid process to the tensor-vascular-styloid fascia, which consists of the *tensor veli* palatine muscle, its fascia, and the stylopharyngeal and styloglossus muscles (4, 7). The anatomical characteristics of the PPS make clinical examination and surgical management complicated (8). Traditional surgical approaches for PPLN tumors involve transcervical and transoral approaches with limited working space and mandibular dissection is sometimes needed.

In 1998, Miccoli et al. incorporated a minimally invasive video-assisted (MIVA) technique into thyroid surgery (9). The MIVA technique has been widely accepted in the management of benign or early malignant thyroid lesions. In 2005, Giordano et al. (10) first described MIVA surgery for two PTC cases with PPLN metastases. Here, based on our previous experiences, we reported a case series of PTC patients with PPLN metastases managed with MIVA technique.

## Materials and Methods

### Patients

From January 2012 to July 2018, six consecutive PTC patients with PPLN metastases in the Department of Thyroid Surgery, the Second Affiliated Hospital of Nanchang University, were enrolled in this retrospective study. Informed consent was obtained from all patients. The Ethics Committee of the Second Affiliated Hospital of Nanchang University approved this study. A single experienced surgeon (Ji-Chun Yu) performed all operations in this study (11, 12).

The following eligibility criteria were applied for the current study: (1) pathologically proven primary PTC with suspicious PPLN metastases shown on preoperative enhanced computed tomography (CT), (2) post-operative follow-up CT scan showing PPLN recurrence, (3) preoperative CT showing PPLN without extranodal extension, (4) patients' preferences, and (5) well-documented medical history. The following exclusion criteria were also applied: (1) patients with previous neck radiation therapy history, (2) patients with previous PPS surgery history, (3) patients who refused to undergo surgery with the MIVA approach, and (4) preoperative CT showing PPLN with extranodal extension.

### Surgical Technique

All patients were operated under general anesthesia by the same surgical team based on a previous study (13).

**Figure 1:**
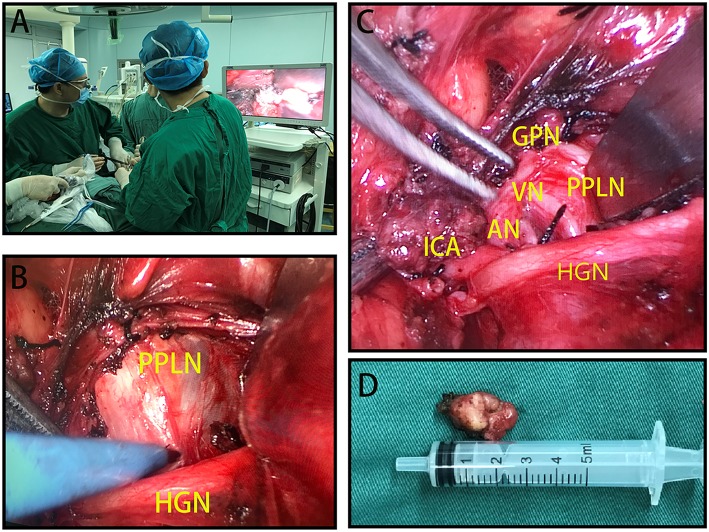
**(A)** Positions of the surgical team when performing minimally invasive video-assisted (MIVA) parapharyngeal space lymph node (PPLN) resection. **(B)** PPLNs located interior to the hypoglossal nerve (HGN). **(C)** PPLNs can be removed, and surrounding structures are carefully exposed and preserved. **(D)** Resected PPLN specimen. HGN, hypoglossal nerve; ICA, internal carotid artery; AN, accessory nerve; VN, vagus nerve; GPN, glossopharyngeal nerve; PPLN, parapharyngeal space lymph nodes.

Surgical treatment for a new diagnosis of thyroid cancer with PPS metastasis involved a total thyroidectomy plus central (level VI) and selective or modified lateral (levels II–V) neck dissection plus lymph node dissection of the PPS. For patients with recurrence only in PPLNs, PPLN resection was performed. Surgical procedures required a hockey stick incision. A traditional open total thyroidectomy and neck dissection was performed, and the parathyroid glands and recurrent laryngeal nerves (RLN) were identified and preserved. Then, a skin flap was raised under the platysma muscle layer continually, and a 0-degree endoscope (Karl-Storz Endoscope, Tuttlingen, Germany) and harmonic scalpel (Ethicon Inc., Somerville, NJ, USA) were used to create a working space. The PPS was exposed by dissecting along the deep surface of the digastric muscle with bipolar coagulation forceps (Hutong, Shanghai, China). Then, the internal borders of the internal and external carotid arteries were exposed and preserved. Subsequently, a retractor was used to retract outwards and protect these vessels; the vagus nerve, accessory nerve, glossopharyngeal nerve, and hypoglossal nerve should be exposed and preserved correctly. Then, the PPLNs were revealed at the inner front of the internal carotid artery and should be removed carefully with bipolar coagulation forceps (Figure 1).

### Post-operative Treatment and Follow-Up

All patients received post-operative RAI therapy. Post-operative complications were also recorded, including permanent or transient RLN paralysis, hypoparathyroidism, palatal weakness, Horner's syndrome, tongue weakness, facial nerve paralysis, limited elevation of the shoulders, first bite syndrome, and cerebrospinal fluid leakage. Post-operative assessments with cross-sectional imaging were performed every 6 months to detect locoregional or distant recurrence. For locoregional recurrence that was surgically resectable, additional surgical treatment was performed.

### Statistical Analysis

Continuous variables are expressed as medians (ranges). Categorical variables are expressed as percentages.

## Results

**Figure 2:**
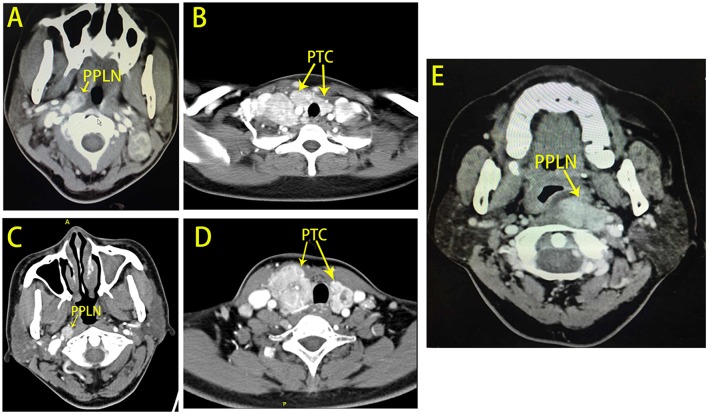
Computed tomography (CT) scans for patients with PPLN metastases. **(A,B)** Show patient No. 3 with bilateral papillary thyroid carcinoma with right PPLN metastases. **(C,D)** Show patient No. 4 with bilateral papillary thyroid carcinoma with right PPLN metastases. **(E)** Shows patient No. 5 with left PPLN recurrence 17 years after the first surgery.

Six patients (three women and three men) who underwent surgery were enrolled in the current study (Table 1). The median age of the patients was 42 years (32–66). Five patients (83.3%) were diagnosed with primary PTC with PPLN metastases, and one patient had PTC recurrence in PPLNs 17 years after her first PTC surgery. All of the patients had suspicious PPLN metastases identified on preoperative enhanced CT (Figure 2). Surgical treatment was successful in all patients, and the median operative time and blood volume were 185 (100–280) min and 85 (30–120) ml, respectively. None of the patients experienced post-operative complications, such as hypoparathyroidism, vocal cord paralysis, Horner's syndrome, tongue weakness, facial nerve weakness, a limited elevation of the shoulder, or other conditions. The median numbers of positive and retrieved PPLNs were 1 (1–2) and 2 (1–2), respectively. Post-operative radioiodine therapy (RAI) was applied in all six patients with a median dose of 100 mCi (30–150). During a median follow-up of 15 months (10–31), none of the patients exhibited recurrence or persistent disease.

**Table 1:** Characteristics and related data of PTC patients with PPLN metastases.

**Case no**.	**Age/** **sex**	**Treatment**	**Positive PPLN/retrieved PPLN**	**PPLN size (cm)**	**Operative time (min)**	**Operative bleeding (mL)**	**Hospital stay (days)**	**Post-** **operative complications**	**Clinical** **staging**	**Post-operative treatment with 131I (mCi)**	**Follow-up (months)**
1	66/M	TT + CND + SND(II–V, R) + PLND(R)	1/1	1.3*1.5*1.0	180	100	14	None	T1N1bM0, I	100	16
2	32/M	TT + CND + SND(II–V, R) + PLND(R)	1/1	2.5*1.5*1.0	220	50	16	None	T1N1bM0, I	100	14
3	39/F	TT + CND + SND(II–V, L&R) + PLND(R)	1/2	2.0*1.5*0.5	280	120	18	None	T2N1bM0, I (bilateral)	100	20
4	54/M	TT + CND + SND(II–V, R) + PLND(R)	1/1	3.0*2.0*1.0	190	80	14	None	T4N1bM0, I	150	10
5	42/F	PLND(L)	2/2	3.2*2.5*1.0	100	30	7	Dysphagia, resolved within 3 months	PPLN recurrence in 17 years after the first surgery	30	31
6	39/F	TT + CND + SND(II–V, L&R) + PLND(R)	1/2	2.1*1.5*0.8	180	90	13	None	T3N1bM0, I (bilateral)	100	12

*TT, total thyroidectomy; CND, central neck dissection; SND, selective neck dissection; PLND, parapharyngeal lymph nodes dissection; L, left; R, right; PPLN, parapharyngeal lymph nodes*.

## Discussion

In the current study, for the first time, we evaluated the safety and feasibility of PPLN dissection via a MIVA approach for six thyroid cancer patients who experienced PPLN metastases. The advantage of this technique allows surgeons to safely remove PPLNs without mandibulotomies or additional incisions and without compromising the surgical view.

The regional lymph nodes most commonly involved in thyroid cancer are the central compartment lymph nodes (level VI), followed by the jugular chain (levels II–IV), the posterior cervical nodes (level V), and the submandibular and submental nodes (level I) (14). PPLN metastases are relatively rare for thyroid cancer upon initial treatment and recurrences. A possible anatomic explanation for this phenomenon has been proposed and attributed to lymphatic drainage from the jugular chain or direct drainage from the upper pole of the thyroid gland followed by draining to the retropharyngeal space. Moritani (6) previously described a lymphatic vessel connecting the upper pole of the thyroid gland to the retropharyngeal lymphatic system. This lymphatic trunk could be observed in 20% of the cases. Dehiscence behind the fascia of the superior constrictor muscle could be observed, making communication between the parapharyngeal and retropharyngeal spaces possible. In our study, all five primary cases presented with tumors located at the upper pole of the thyroid gland (including one recurrence case whose primary tumor was located at the upper pole of the thyroid as well).

PPLN metastases present few symptoms and may be associated with dysphagia, cranial nerve deficits, Horner's syndrome, pain, and hoarseness (6). Of the six thyroid cancer patients with PPLN metastases, only two patients (33.3%) exhibited symptoms. Therefore, there is a challenge in diagnosing PPLN metastases based on clinical signs. In our series, all PPLN metastases were identified by enhanced CT. Some reports have suggested that MRI or neck enhanced CT should be considered in post-thyroidectomy patients with increased serum thyroglobulin levels and those with cervical metastasis in the jugular region (15–17). Metastatic PPLNs have several distinct imaging features. PPLNs often present at the posterior parapharyngeal space and the internal jugular vein as well as the internal carotid artery located at the posterior or post-erolateral aspect of the PPLNs. The morphological features of PPLNs are consistent with other metastatic lymph nodes in other parts of the neck, including calcification, degrees of enhancement, liquefaction, and a generally intact lymph node capsule. These characteristics contribute to the differential diagnosis in other tumors in the parapharyngeal space. Salivary gland-derived tumors locate at the pre-styloid parapharyngeal space. Nerve-derived tumors locate at the post-styloid parapharyngeal space but locate posterior to the carotid sheath, pushing these vessels anteriorly and laterally. Enhanced-CT scan showed uneven density and cystic necrosis with a clear boundary. PPLNs from anaplastic or poorly differentiated thyroid cancers also surround the carotid sheath, with CT scans showing lymph node features, including apparent necrosis, extranodal extension, and shared common features with their primary sites.

Traditional management of PPLN metastases has involved transoral and transcervical, or transcervical–transparotid approaches, with or without several mandibulotomies (18). The anatomical shape of the PPS has always highlighted the problem of improved exposure for a safer and complete excision. However, transoral and transcervical approaches may increase surgical risk due to the relatively small working space. Moritani (6) reported several complications in parapharyngeal space surgery via a transcervical approach, including 11% of patients with palatal weakness, 2% with Horner's syndrome, 2% with tongue weakness and facial nerve weakness, and 1% with limited elevation of shoulders. Recently, transoral robotic surgery for PPLN metastases has been introduced, indicating that this instrument may play an essential role in the future (19). However, this technique has some limitations preventing it from becoming widely accepted, including increased expenditure and a lack of force feedback. In our experience, transcervical MIVA is a safe and feasible approach for patients with PPLN metastases. Only one patient had dysphagia, which resolved within 3 months post-operatively. No patients experienced post-operative complications. Endoscopy can provide a magnified and more unobstructed view in a narrow working space; therefore, a mandibular osteotomy is not necessary for our approach. It also allows safe preservation of the marginal branch of the facial nerve and the inferior alveolar nerve and avoids scarring and facial deformities.

During the operation, several tips may be of use. First, hemostasis is critical, and external carotid artery ligation should be applied when necessary. If bleeding is difficult to control, including upper internal jugular vein hemorrhage, a mandibular osteotomy should be performed for better exposure. Second, PPLNs are located along the arterial sheath; therefore, vagus and sympathetic nerves are vulnerable to sacrifice. Our experience is that the nerves should be exposed carefully before resecting the metastatic lymph node. Third, metastatic PPLNs may have intact capsules, which can be separated from the skull base jugular foramen. Therefore, cerebrospinal fluid leakage can occur. When upper internal jugular vein bleeding occurs, clamping and tearing may cause cerebrospinal fluid leakage, which presents as a clear liquid leaking from the skull base. Our suggestion for the management of this complication is as follows: first, complete hemostasis is necessary, and a fat tissue flap should then be used to fill the skull base.

Our technique may have some limitations. First, based on our experiences, if metastatic lymph nodes are >4 cm in diameter, open surgery is recommended for a safe procedure. Second, if preoperative imaging shows that the metastatic lymph nodes invade the internal carotid artery or gross extranodal extension, MIVA is a contradiction for these patients; thus, open surgery is recommended. There are several limitations to the current study. First, the retrospective nature of this study may have an inevitable bias. Second, future comparative studies for an open and MIVA approach are required to further evaluate the safety and feasibility of our approach.

## Conclusion

The MIVA transcervical approach is technically feasible and reliable, with less invasiveness for PTC patients with PPLN metastases. This approach should be popularized based on its practicality and low surgical invasiveness compared with robot-assisted surgery and other surgical procedures. However, future studies are needed to accumulate more experience and to investigate the indications of the technique as well as to determine the long-term oncological safety.

## Data Availability Statement

The datasets generated for this study are available on request to the corresponding author.

## Ethics Statement

The studies involving human participants were reviewed and approved by Ethics Committee of the Second Affiliated Hospital of Nanchang University. The patients/participants provided their written informed consent to participate in this study.

## Author Contributions

S-TY, J-CY, and W-ZC conceived and designed the study. TZ organized the database. S-TY performed the statistical analysis and wrote the first draft of the manuscript. D-BX, W-ZC, and TZ wrote sections of the manuscript. All authors contributed to manuscript revision and read and approved the submitted version.

### Conflict of Interest

The authors declare that the research was conducted in the absence of any commercial or financial relationships that could be construed as a potential conflict of interest.
